# Myoelectric control and virtual reality to enhance motor rehabilitation after stroke

**DOI:** 10.3389/fbioe.2024.1376000

**Published:** 2024-04-11

**Authors:** Denise Jennifer Berger, Andrea d’Avella

**Affiliations:** ^1^ Laboratory of Neuromotor Physiology, IRCCS Fondazione Santa Lucia, Rome, Italy; ^2^ Department of Systems Medicine, Centre of Space Bio-medicine, University of Rome Tor Vergata, Rome, Italy; ^3^ Department of Biology, University of Rome Tor Vergata, Rome, Italy

**Keywords:** stroke, motor rehabilitation, myoelectric control, virtual reality interface, rehabilitation technology

## Abstract

Effective upper-limb rehabilitation for severely impaired stroke survivors is still missing. Recent studies endorse novel motor rehabilitation approaches such as robotic exoskeletons and virtual reality systems to restore the function of the paretic limb of stroke survivors. However, the optimal way to promote the functional reorganization of the central nervous system after a stroke has yet to be uncovered. Electromyographic (EMG) signals have been employed for prosthetic control, but their application to rehabilitation has been limited. Here we propose a novel approach to promote the reorganization of pathological muscle activation patterns and enhance upper-limb motor recovery in stroke survivors by using an EMG-controlled interface to provide personalized assistance while performing movements in virtual reality (VR). We suggest that altering the visual feedback to improve motor performance in VR, thereby reducing the effect of deviations of the actual, dysfunctional muscle patterns from the functional ones, will actively engage patients in motor learning and facilitate the restoration of functional muscle patterns. An EMG-controlled VR interface may facilitate effective rehabilitation by targeting specific changes in the structure of muscle synergies and in their activations that emerged after a stroke—offering the possibility to provide rehabilitation therapies addressing specific individual impairments.

## 1 Introduction

Over 85% of stroke patients suffer from functional deficits in motor control (Langhorne et al., 2011). As upper-limb recovery is essential for regaining functional independence, there is growing interest in developing technologies that enhance rehabilitation outcomes for stroke survivors. While Ward et al. (2019) found that intensive rehabilitation has high recovery potential in stroke survivors, other traditional rehabilitation treatments failed to demonstrate long-term efficacy for functional recovery (Bell et al., 2015; Wu et al., 2016). The former study did not include a control group -- that did not receive additional intensive therapy -- to evaluate intensive therapy against spontaneous recovery. Taken together, innovative methodologies are needed. Recent studies endorse the use of therapies that use exoskeletons (Hohl et al., 2022), brain-machine interfaces (Ramos-Murguialday et al., 2013; 2019), and VR devices (Vourvopoulos et al., 2019; Qiu et al., 2020; Fluet et al., 2021) to restore the function of the paretic limb after stroke (Stinear et al., 2020; Vidaurre et al., 2023; Amin et al., 2024). These technologies provide new opportunities in rehabilitation as they stimulate intense practice or engage patients in immersive exercises. Although these approaches offer an environment in which the many variables that influence motor behavior can be controlled and show great potential for motor rehabilitation, we know little about what aspects of training are more effective in promoting functional reorganization of the central nervous system (CNS) (Vidaurre et al., 2023; Amin et al., 2024).

Although it is widely accepted that a large number of active movement repetitions are required to induce neuronal changes, a specific number of active movements has not been defined (Grosmaire et al., 2022) and unlike conventional therapy, robotic devices can provide hyper-repetitive therapy at a reasonable cost (Blank et al., 2014) and without excessive fatigue. Nevertheless, many robot-assisted rehabilitation programs may not encourage (enough) active participation at the level necessary to promote neural reorganization in stroke patients (Marchal-Crespo and Reinkensmeyer, 2009), as in many cases voluntary motor intention is not necessary for movement assistance (Song et al., 2013). In contrast, myoelectric-controlled approaches, such as EMG-triggered (Dipietro et al., 2005) and EMG-controlled robot assistance (Loconsole et al., 2014) are specifically designed to enhance active participation. However, EMG signals have been used mostly for controlling prosthetic devices (Farina et al., 2014)—their applications to robot-aided and VR rehabilitation are often ineffective for severely impaired patients. Consequently, severely affected stroke survivors have minimal treatment options and often remain severely disabled (Byblow et al., 2015; Winters et al., 2015).

Recently, some research groups have started to use myoelectric signals as inputs to rehabilitation interfaces intended to restore function. Sarasola-Sanz et al. (2018) hypothesized that using myoelectric control of the pathological EMG signals from the paretic limb of a stroke survivor would likely lead to pathological motion. Hence, they aimed at using EMG signals from the healthy limb and mirroring the signal to assist movements of the paretic limb. Despite its potential, the authors could apply this approach successfully only to healthy subjects, but not to stroke survivors, as they typically showed poor task performances.

In another recent study, Roh and others developed a myoelectric computer-interface training program that reduces abnormal co-activation of upper-limb muscle pairs (Seo et al., 2022). Participants were trained on muscle pairs whose activations were mapped onto the motion of a cursor along the cardinal axes only if one of the two muscles were activated. The authors showed that myoelectric training effectively reduced abnormal co-activation between muscle pairs in stroke survivors. A limitation of this approach is that the use of EMG-control is limited to muscle pairs and the mapping between muscle activations and cursor directions does not reflect the physiological mapping between muscle activation and force directions.

Despite the potential of these personalized EMG-control strategies, the degraded performances motivate the need for more accurate prediction of motion from EMG signals in individuals in a post-stroke rehabilitation setting. To this end, we believe that it is possible to go one step further in the use of EMG-control for rehabilitation by both including a large number of muscles and relying on the physiological mapping between muscles and forces. We aim to facilitate the restoration of functional muscle patterns, not just individual muscles. To this end, we propose to use an EMG-controlled isometric VR interface. EMG-control offers the possibility of delivering intuitive visual feedback about the effectiveness of muscle activity and assisting the patients by altering the visual feedback (virtual assistance), thus reducing the effect of the deviations of the actual, dysfunctional muscle patterns from the functional ones. We hypothesize that operating on the visual feedback will promote the re-learning of functional muscle patterns, thus helping patients improve their movement performance. To achieve this, we further propose to exploit the theoretical knowledge that we and other groups have gained about the coordinated activations of muscle groups, i.e., muscle synergies (Bizzi et al., 2008; Bizzi and Cheung, 2013). Several studies have quantified upper-limb synergies after stroke (Cheung et al., 2009; 2012; Berger et al., 2017; Funato et al., 2022). Muscle synergies were shown to be preserved after a mild stroke in the affected arm, although abnormal muscle activations could be observed. In more severely impaired individuals, the synergy similarity between the affected and unaffected arms was much weaker. Synergies in the affected arm, however, could be derived by merging some of the synergies of the unaffected arm, and the amount of merging was related to the severity of the motor impairment (Cheung et al., 2009; 2012). Moreover, in some individuals, synergies in the affected arm appeared to be fractionations of the synergies in the unaffected arm, and the amount of fractionation increased with stroke duration. These studies demonstrated that motor impairments in stroke survivors can be understood as impairments in synergies (for severely affected individuals) and impairments in synergy activations (for mildly affected individuals). In this view, a mild stroke may impair movements due to dysfunctional synergy activations, and a more severe stroke might be attributed to an altered structure of the synergies (abnormal or dysfunctional synergies).

## 2 Virtual surgeries

**FIGURE 1:**
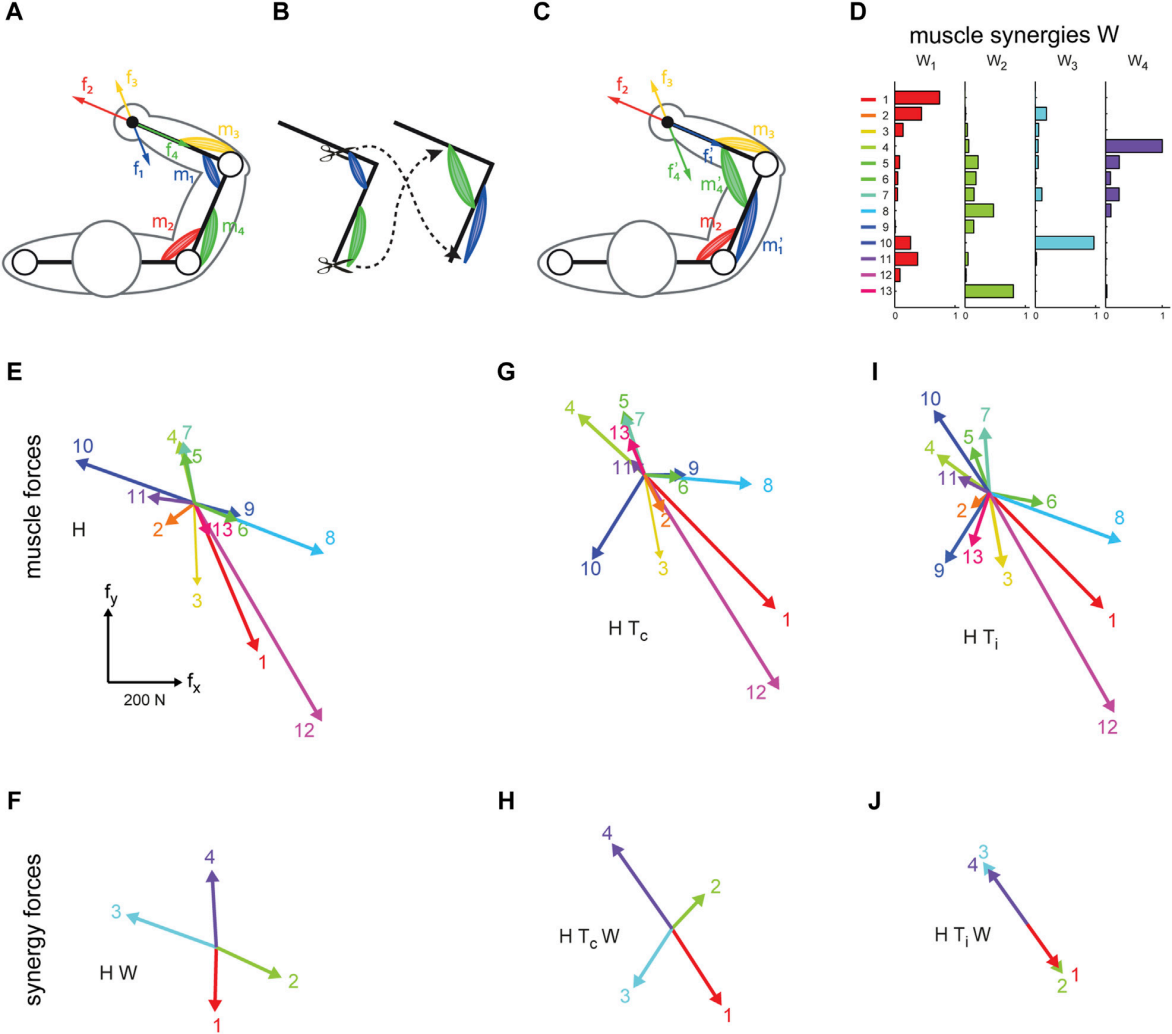
Illustration of virtual surgery concept (modified from Berger et al., 2013). **(A)** Conceptual arm with two muscle pairs, each generating a force in a specific direction at the end point (f1 to f4). **(B)** A tendon transfer surgery affecting the force generated by two muscles. **(C)**, After the surgery, both muscles generate forces in another direction. **(D–J)**: Examples of muscle synergy (W), an EMG-to-force matrix (H matrix), synergy forces (HW), virtual surgeries (HT) and their corresponding projection in the synergy space (HTW) extracted for one healthy subject. **(D)** Spatial muscle synergies (matrix W) are identified by nonnegative matrix factorization from the EMG data. **(E)** Each column of H, represents a pulling force of one muscle. **(F)** Synergy forces span the entire force space. **(G)** Forces generated by muscles after a compatible virtual surgery obtained by recombination of the original forces as after a complex rearrangement of the tendons (Tc). **(H)** Synergy forces after the compatible surgery still span the force space. **(I)** Muscle forces after an incompatible surgery. **(J)** Such rotation aligns the forces associated with the synergies in the same dimension; such that they do not span the entire force space.

We have developed a VR interface that allows participants to control a virtual cursor under the effect of isometric forces recorded or estimated in real-time from EMG recordings, either generated by each muscle (EMG-control, EC) (Berger et al., 2013) or by each muscle synergy (Synergy-control) (Berger and d’Avella, 2014). Myoelectric control in an EC-VR interface allows the simulation of motor dysfunction in healthy human subjects by manipulating the mapping between muscles and end-effector forces (EMG-to-force mapping). By modifying the EMG-to-force mapping we simulated complex surgical modifications of the upper-limb musculoskeletal system, or *virtual surgeries* (see Figure 1 for a schematic example of virtual surgery and an example of their effects on muscle and synergy forces). When these simulated modifications were incompatible with subject-specific muscle synergies, they provoked failures in learning new muscle patterns required to overcome the perturbation. This approach was novel in two respects: it was the first attempt to characterize and quantify motor adaptation to different types of virtual surgeries (compatible or incompatible with the subject-specific synergies). In addition, it was the first attempt to relate failure in learning to muscle synergies. We showed that failure of adaptation is related to the nature of the virtual surgery and demonstrated that synergies determined the feasibility of motor learning. However, just like learning a novel motor skill, it is possible to learn how to perform a reaching task after an incompatible perturbation with enough practice. Indeed, increasing the time allowed for task-space exploration, participants improved their task performance also after incompatible surgeries—synergies are not hard-wired but may be learned with sufficient practice (Berger et al., 2022a). Moreover, we found persistent changes in the muscle patterns following the exposure to an incompatible perturbation, after the re-adaptation to the baseline mapping, and after a subsequent virtual surgery that is compatible with the subjects’ muscle synergies (Berger and d’Avella, 2023a; Berger and d’Avella, 2023b).

Synergy control proved to be a valid control mechanism potentially employed by the brain, as human subjects are immediately able to perform a task after switching from force-control to EMG-control and Synergy control, without significant differences in performances between the control modes (Berger and d’Avella, 2014). Importantly, the switch from one control mode to another was always performed without the subject’s awareness. Synergy control mimics the natural control patterns and demonstrates that muscle synergies can be used as effectively as individual muscles to control cursor movements, showing moreover that muscle synergies provide an effective strategy for motor coordination.

## 3 Assistive control in VR may enhance motor recovery after stroke

Motor impairment in stroke survivors is characterized by abnormal muscle activation patterns. By identifying muscle pattern changes following a stroke, it may be possible to develop personalized rehabilitation methods, specifically addressing the individual impairments. We hypothesize that providing information on the impaired motor control strategies and the underlying dysfunctional neural processes might be critical to drive adaptive plasticity. As visual feedback during movements plays an important role in motor learning (Krakauer et al., 2019), we suggest that by providing salient information about the activation of specific abnormal muscle patterns via visual feedback, patients may learn to regain control of their muscular activity and to recover functional muscle activation patterns. Research on motor learning has emphasized that errors are needed in order to drive motor adaptation in healthy subjects (Emken and Reinkensmeyer, 2005) and in stroke survivors (Patton et al., 2006; Reisman et al., 2013). However, augmenting errors did not always cause more effective motor learning when errors were large (Sharp et al., 2011; Marchal-Crespo et al., 2017). We therefore suggest assisting the patients’ voluntary attempt to perform movements in the EC-VR, reinforcing thereby the components of functional muscle patterns by correcting in VR the effect of pathological muscle patterns of the affected upper-limb. We hypothesize that the combination of voluntary movement attempts and feedback from successful movements in VR will increase neural plasticity and enhance functional recovery. The key ingredient is the ability to alter visual feedback according to the recorded muscle activity in real-time, for which a virtual environment is required. Manipulation of the virtual hand’s motion using the recorded muscle patterns enables the experimenter to correct the effect of deviations of the actual (dysfunctional) from a reference (functional) muscle pattern. Specifically, by manipulating the cursor motion associated with the recorded muscle patterns, i.e., using the patient-specific impairments, allows for patient-tailored virtual assistance. It moreover allows the patients to regain control of their muscle activity by receiving feedback derived from their EMG signals, recorded from many muscles, that are informative and provide functional significance, despite their motor impairments.

During passive control when the impaired limb moves along the pre-defined trajectory, active participation is not encouraged, and thus it does not stimulate motor function recovery (Hogan et al., 2006). In order to stimulate the voluntary, active participation of patients, which is necessary for inducing cortical reorganization, we propose to gradually reduce the virtual assistance as performance improves. This will ensure that the task remains feasible but challenging enough to maintain motivation. The visual feedback of the salient errors in the task space will drive adaptation of the dysfunctional muscle patterns and enhance motor recovery. As most of motor recovery occurs in the first 3 months after stroke (e.g., Zeiler and Krakauer, 2013), we expect motor recovery to be enhanced at the early subacute stage (Stinear et al., 2020).

## 4 Assistive, adaptive control algorithm as a novel rehabilitation approach for stroke rehabilitation

We aim at facilitating motor recovery by providing patient-tailored virtual assistance. Here, we present an assistive-adaptive control algorithm that allows patients to control a virtual cursor as if they were generating better-coordinated muscle patterns. Such improvement is achieved by projecting the recorded muscle patterns onto functional muscle space manifolds, which helps maintain high motivation for intensive training. Moreover, by providing salient feedback on the direction in which muscle patterns must be adapted to improve motor function, the control algorithm will amplify residual functional muscle activation capability and guide re-learning of functional muscle patterns.

### 4.1 Assistance during EMG-control

**FIGURE 2:**
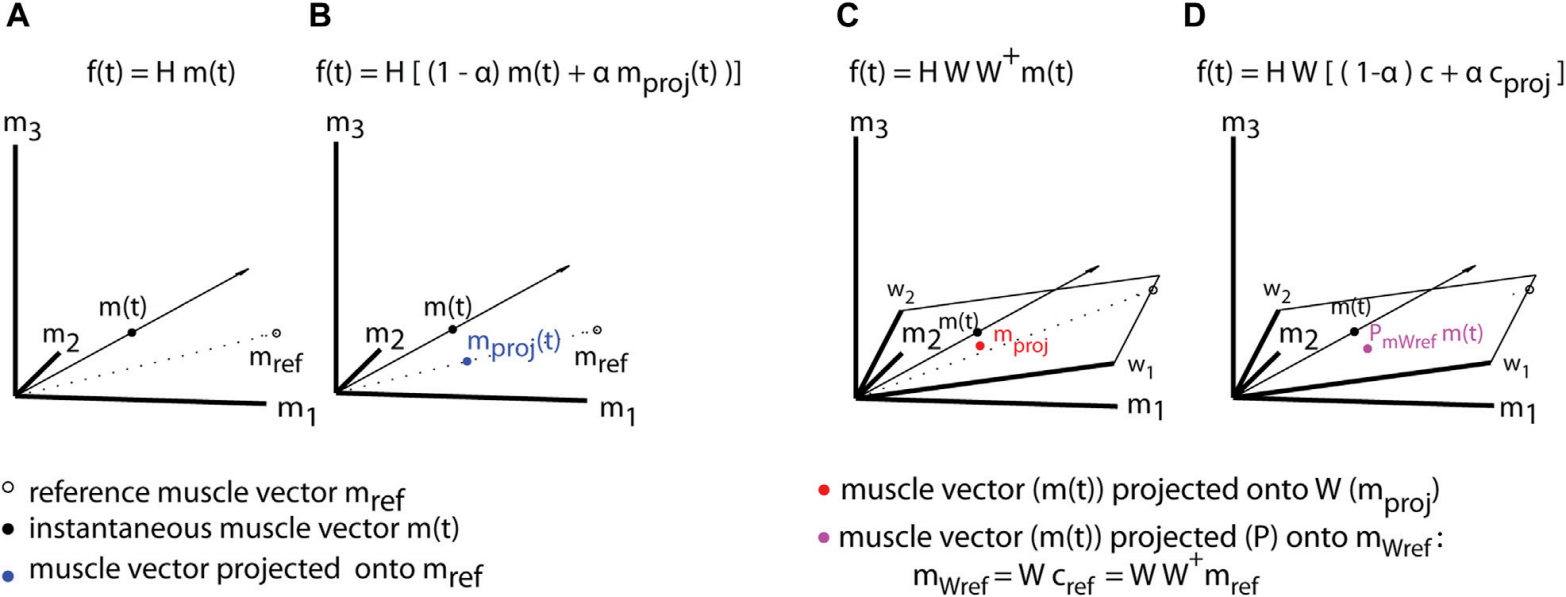
Assistive myoelectric control **(A,B)** and assistive synergy-control **(C,D)**. To assist the movement toward a specific goal, a target-specific reference muscle vector (m_ref_) **(A)** is computed and used to project the instantaneous muscle activity vector (m(t)) onto its direction in muscle space $m_{\text{proj}}=m_{\text{ref}}\;{m_{\text{ref}}}^{T}\;m$ **(B)**. The level of assistance can be regulated by adjusting the parameter α, ranging from 1 (full assistance) to 0 (no assistance). Additional scaling in amplitude along the reference vector may be added to enhance the visual feedback. **(C)** During Synergy-control the recorded muscle activity (m(t)) is projected onto the muscle synergy space W (Berger and d’Avella, 2014). **(D)** Assistance is provided by projecting the instantaneous muscle activations onto synergy manifolds that capture the physiological muscle patterns. The instantaneous synergy coefficients **(C)** are projected onto the reference coefficients ( $c_{\text{ref}}$ ) $c_{\text{proj}}=c_{\text{ref}}{c_{\text{ref}}}^{T}\;c,$ where $c_{\text{ref}}$ is given by $c_{\text{ref}}=W^{+}m_{\text{ref}}$ . As during EMG-control assistance, the level of assistance (α) will be continuously adjusted during training to maintain the participants motivation high and task difficulty constant. To enhance the visual feedback, additional amplitude scaling along the reference vector may be added.

During EMG-control, virtual assistance is provided by correcting in real-time the effect of a deviation of the actual (dysfunctional) recorded muscle activity ***m*** from a reference (functional) muscle pattern ***m***_***ref***_ for a specific force target (f*). The target-specific muscle activity of the unaffected limb or the average of the activity of a set of healthy subjects serves as the reference muscle activity. More specifically, the forces generated by the patient (***f***_***real***_) can be expressed as ***f***_***real***_ = ***Hm*** and the recorded muscle activity of the paretic limb is projected in real-time onto the task-specific reference functional muscle pattern:

$$m_{proj}=m_{ref}\;{m_{ref}}^{T}\;m$$

such that the projected muscle pattern directs the cursor in the correct target direction. However, the force f acting on the cursor in VR is determined as a weighted combination of the force generated by the actual muscle pattern (***m***) and the muscle pattern projected on the reference pattern:

$$f=H\;\left[\;\left(\;1-\alpha \;\right)m+\alpha \;m_{proj}\;\right]$$

with α being the amount of assistance and ***H*** is the EMG-to-force mapping that relates the muscle activations to cursor forces in the virtual environment. Figure 2 (A-B) shows how a muscle template for a specific goal is constructed ( $m_{ref}$ ), and how the actual, potentially dysfunctional muscle activation pattern ***m*** may be corrected by projecting it onto the reference muscle pattern. The assistive force allows the patient to move the virtual hand in the correct direction. By reinforcing the components of the patient’s muscle activations closer to the physiological ones we expect to minimize abnormal co-contraction patterns and to favor physiological and more functional movements. For each patient, the appropriate feedback is designed according to their muscle effort, with a reinforcement of the more efficient motor strategies that the patient is free to explore. The EMG-to-force mapping will be estimated from upper-limb muscle activity of the unaffected arm or of a set of healthy subjects. Since the task will be performed in isometric conditions, the generated forces are approximately a linear function of muscle activations**,** and the EMG-to-force mapping is estimated using multiple linear regressions of each applied force component with EMG signals and recorded forces. In such a way we create a model for the EMG-to-force mapping that can then be used for the paretic arm.

The level of assistance can be adjusted during training. With full assistance $\left(\alpha =1\right)$ the cursor moves along the target direction as if the muscles were activated correctly. To stimulate the voluntary, active participation of the patients, the level of assistance will be gradually reduced according to the patient’s performance, ensuring an optimal balance between task feasibility and difficulty, in such a way that the task difficulty is maintained invariant throughout the rehabilitation procedure. As the level of assistance decreases, the cursor motion deviates from the intended direction but provides salient feedback on the changes required in the muscle patterns to regain correct performance. Thus, our VR environment provides the patients with salient, visual feedback about their muscle activations, which they have to actively integrate to move the cursor toward the target position, thereby stimulating the recruitment of the appropriate muscle patterns.

### 4.2 Assistance during synergy-control

Muscle synergies have been proposed as a way used by the CNS to simplify the generation of motor commands by decreasing the redundancy of the muscular system. The interpretation of abnormal muscle activations in patients in terms of muscle synergies would allow to choose corrective actions within the functional muscle patterns that maximize movement efficiency. In such a way, only the dysfunctional part of the muscle pattern is corrected, while the functional part is rewarded as it allows to successfully complete the movement.

We therefore suggest as a next step to use synergy-based adaptive control for upper-limb virtual assistance. This approach exploits the fact that the modular architecture of the CNS may provide a framework that opens the possibility of enhancing EC/VR-steered motor recovery which has the potential to selectively facilitate the restoration of specific components of the CNS that have been lost.

During Synergy-control the recorded muscle pattern is projected onto the task-specific synergy space (Figure 2C). This approach assists by filtering out those components of the muscle activation, such as abnormal muscle co-activation, that are not captured by physiological (functional) synergies. The projection onto a functional, reference synergies will allow the patients to improve motor performance, but it will require their active participation. Virtual assistance during Synergy-control is provided by correcting in real-time the effect of a deviation of the dysfunctional (actual) synergy coefficients ***c*** (online-decoded) from the functional (reference) coefficients ***c***_***re*f**_ for a specific target (f*). A detailed description of online-decoded muscle synergies and synergy coefficients is given in (Berger and d’Avella, 2014). The projection of the actual synergy coefficients onto the reference coefficients:

$$c_{proj}=c_{ref}{c_{ref}}^{T}\;c$$

where the reference coefficients will be computed as:

$$c_{ref}=W^{+}m_{ref}$$

where ***W*** ^***+***^ denotes the pseudoinverse of the matrix of the functional, reference muscle synergies.

The force f is then determined by:

$$f=H\;W\;\left[\left(\;1-\alpha \;\right)c+\alpha \;c_{proj}\right]$$

Functional, reference muscle synergies, the EMG-to-force mapping, and target-specific coefficients are estimated from the unaffected limb or healthy subjects (Figure 2D). The amount of assistance (α) will be continuously adjusted during training to maintain a high level of engagement and a constant task difficulty. We suggest that learning of novel, functional synergies could be enhanced with such a procedure that promotes the development of new functional synergy recruiting by virtual assistive control.

## 5 Discussion

We propose that an EMG-controlled VR interface that actively engages patients in motor learning has a high potential to enhance upper-limb rehabilitation after stroke. This novel rehabilitation approach provides subject-specific and performance-dependent virtual assistance, implemented by modifying the EMG-to-force map that in turn alters the visual feedback the patients receive. By correcting the components of the dysfunctional muscle patterns and providing salient and informative feedback on the functional components of the motor output, performance is improved, and neural plasticity increases, enhancing motor recovery.

While many approaches to motor rehabilitation after stroke have been developed very few have targeted muscle activation patterns. Electromyographic biofeedback of single muscles had mixed results (Wolf, 1983; Schleenbaker and Mainous, 1993), and largely sought to strengthen muscles or reduce spasticity. Several studies have used EMG feedback to change the co-activation of agonist and antagonist muscles during training in stroke participants (Mugler et al., 2019; Jian et al., 2021; Marin-Pardo et al., 2021). Seo et al. (2022) found a significant change in the co-contraction of targeted muscle pairs. However, all these studies have in common the use of muscle pairs only—they did not consider the high dimensionality of muscle coordination.

Motor impairments in stroke survivors have been associated with disruption of synergies (dysfunctional synergies for severely affected stroke survivors) or synergy activations (for mildly affected stroke survivors, Cheung et al., 2009; 2012). If motor recovery after stroke is dependent on the re-organization of the dysfunctional synergy structure and re-acquisition of appropriate synergy recruitment, characterization of such modular organization and identification in the synergy structure and/or synergy activations may allow for an objective and reliable assessment of functional impairment and efficient therapeutic in stroke survivors. However, only very few, more recent studies, have looked into the effects of stroke rehabilitation and its potential to change dysfunctional muscle synergies (Hong et al., 2021; Seo et al., 2022).

To fully assess the effectiveness of personalized myoelectric control and to better understand the adaptive processes and their neural implementation, additional experiments with patients, possibly combined with a stimulation protocol are required. Related to this, muscle synergy changes related to specific lesions would provide a novel validation of modularity in the motor system (Berger et al., 2020; 2022b).

Our EMG-controlled interface is novel in that it considers a large number of muscles and makes the mapping between muscle activity and task space such that the virtual hand moves according to muscle activity patterns that match the actual isometric forces of healthy human subjects. This leads to the reasonable expectation that the training with synergy-based myoelectric control of a virtual hand, selectively stimulating the restoration of functional synergy recruitments, would significantly improve further recovery after stroke. Our approach fully exploits the hypothesis of a synergistic organization of the neural control of movement. This approach has the potential to provide a new framework for neurorehabilitation interventions, as it opens the possibility that motor recovery might be enhanced with innovative VR tools and EMG-control that can selectively stimulate the restoration of functional synergy organization and synergy recruitment. Our assistive algorithm may stimulate intensive, personalized training and promote active participation of the patient with a tool that relates directly to the specific pathophysiology.

Many studies have shown that motor learning is an error-driven process (Abdollahi et al., 2014; Liu et al., 2018). While the theories that support error reduction and error augmentation paradigms are distinct, it is still an open issue as to which of the two paradigms provides superior treatment effects in upper extremity motor recovery and performance among stroke survivors (Sharp et al., 2011; Liu et al., 2018). As a next step we suggest using the error augmentation paradigm instead of the error reduction paradigm as done by, e.g., Patton et al. (2006) and Abdollahi et al. (2014). Assistive therapy might be more effective in the initial stage of motor learning in the first few months after a stroke, while error-based learning is more effective in the chronic stage (Liu et al., 2018). Indeed, it has been shown that in the initial stage of motor learning, motivation, and positive reinforcement are believed to be more important than error identifications (Sidarta et al., 2016).

Future work will address the potential neural mechanisms underlying the hypothesized enhancement of upper-limb stroke rehabilitation provided by virtual assistance. To better understand how the reduction of visual error provided by virtual assistance could be used by a patient to infer needed corrections in the muscle patterns we need to model the generation of dysfunctional muscle patterns in a synergy-based controller and the adaptive processes underlying motor recovery post-stroke. Future work should moreover address the substitution of simple visual feedback to haptic feedback, e.g., actual arm motion of the paretic limb using a myoelectric-controlled exoskeleton. We furthermore suggest that precisely-timed TMS pulses over the motor cortex during movement planning could further enhance the neuroplasticity.

## Data Availability

The original contributions presented in the study are included in the article/Supplementary material, further inquiries can be directed to the corresponding author.
